# Epinephrine affects gene expression levels and has a complex effect on biofilm formation in *Micrococcus luteus* strain C01 isolated from human skin

**DOI:** 10.1016/j.bioflm.2021.100058

**Published:** 2021-10-21

**Authors:** A.V. Gannesen, M.I. Schelkunov, O.V. Geras'kina, N.E. Makarova, M.V. Sukhacheva, N.D. Danilova, M.A. Ovcharova, S.V. Mart'yanov, T.A. Pankratov, D.S. Muzychenko, M.V. Zhurina, A.V. Feofanov, E.A. Botchkova, V.K. Plakunov

**Affiliations:** a Federal Research Center “Fundamentals of Biotechnology” of the Russian Academy of Sciences, Moscow, Russia; b Skolkovo Institute of Science and Technology, Moscow, Russia; c Institute for Information Transmission Problems, Moscow, Russia; d Faculty of Biology, Lomonosov Moscow State University, Moscow, Russia; e Shemyakin-Ovchinnikov Institute of Bioorganic Chemistry, Russian Academy of Sciences, Moscow, Russia

**Keywords:** *Micrococcus luteus*, Epinephrine, Biofilms, RNA-seq, Transcriptomics, Confocal microscopy, Skin microbiota, Human-microbiota interaction, qPCR

## Abstract

In this study, the effect of epinephrine on the biofilm formation of *Micrococcus luteus* C01 isolated from human skin was investigated in depth for the first time. This hormone has a complex effect on biofilms in various systems. In a system with polytetrafluoroethylene (PTFE) cubes, treatment with epinephrine at a physiological concentration of 4.9 × 10^−9^ M increased the total amount of 72-h biofilm biomass stained with crystal violet and increased the metabolic activity of biofilms, but at higher and lower concentrations, the treatment had no significant effect. On glass fiber filters, treatment with the hormone decreased the number of colony forming units (CFUs) and changed the aggregation but did not affect the metabolic activity of biofilm cells. In glass bottom plates examined by confocal microscopy, epinephrine notably inhibited the growth of biofilms. RNA-seq analysis and RT–PCR demonstrated reproducible upregulation of genes encoding Fe–S cluster assembly factors and cyanide detoxification sulfurtransferase, whereas genes encoding the co-chaperone GroES, the LysE superfamily of lysine exporters, short-chain alcohol dehydrogenase and the potential c-di-GMP phosphotransferase were downregulated. Our results suggest that epinephrine may stimulate matrix synthesis in *M. luteus* biofilms, thereby increasing the activity of NAD(H) oxidoreductases. Potential c-di-GMP pathway proteins are essential in these processes.

## Highlights

• Physiological blood concentration of epinephrine affected *M. luteus* C01 biofilms.
• Epinephrine stimulated biofilm growth assessed with crystal violet.
• Epinephrine affected the CFU and cell aggregation depend on incubation time.
• 4.912 × 10^−9^ M epinephrine changed the biofilm biomass assessed by CLSM.
• RNA-seq and RT-qPCR showed altered expression levels in seven *M. luteus* C01 genes.

## 1 Introduction

The skin microbiota is a complex community of microorganisms containing hundreds of species. Among these microbes, the phylum Actinobacteria is one of the most abundant [1], and *Micrococcus luteus* is an important component of this community [2,3]. Multispecies biofilms are the most prevalent life form of microorganisms [4], and skin microbiota appears to be no exception.Cutaneous bacteria form biofilms in different skin microniches, such as sweat, sebaceous glands, and hair follicles [[5], [6], [7]]. Although *M. luteus* is a ubiquitous microorganism that was discovered more than a century ago and is also detected in soil and water [8], its biofilms have not been thoroughly investigated to date. Most likely, this oversight occurred because of the relative safety of this species to human health; only a few cases of *M. luteus*-caused disorders have been reported, [[9], [10], [11]]. Despite its safety, in complex multispecies biofilms in the skin microniches of sebaceous glands, hair follicles, and mucosae, *M. luteus* may serve as an initial colonizer and surface mediator for other, potentially more dangerous microorganisms [12] and a number of other microorganisms, such as the aquatic microbes *Sphingomonas natatoria* and *Brevundimonas lenta* [13,14].

Many compounds synthesized by human cells affect the microbiota, such as various humoral factors, hormones and neurotransmitters [[15], [16], [17], [18]]. Biofilms are resistant to various antimicrobial agents, such as antibiotics, biocides and the host immune system/These properties are important in chronic infectious diseases [19,20], and skin biofilms are not an exception. Thus, it would be useful to searchnovel compounds with antibiofilm activities that do not cause resistance in pathogens. Hormones and other humoral regulators without direct antimicrobial effects have been suggested to be useful as components of future complex antimicrobials, where they could serve as pathogen attenuators. For instance, local application of such an agent in an area of skin inflammation in combination with an antibiotic could increase the antimicrobial effect [21].

Catecholamines norepinephrine, epinephrine, and dopamine are the most intensively studied hormones in the context of humoral-microbial interactions. The first evidence that catecholamines can influence not only human cells but also microorganisms was published in 1992 [22] and represented the basis of a new concept, “microbial endocrinology” [23]. Although there are many data regarding the impact of catecholamines on some gram-negative bacteria, especially *Escherichia coli* [[24], [25], [26]]; [27], less is known concerning their impact on gram-positive bacteria, especially Actinobacteria – a phylum of high abundance on the skin [1]. Recent work was devoted to the impact of epinephrine and norepinephrine on biofilms of the opportunistic skin pathogen *Cutibacterium acnes*. It was found that epinephrine and norepinephrine increased the biofilm growth of *C. acnes* and changed a number of parameters of the bacterium [18] at a concentration of 10^−6^ M. Authors suggest the histidine kinase KdpD (a partial homolog of QseC in *E. coli*) is a probable sensor of epinephrine in *C. acnes*.

In this study, we decided to study the influence of epinephrine on biofilms of *M. luteus*, for several reasons. First, the biofilms of this bacterium have not been thoroughly investigated to date, and it is an important part of the skin microbiota [2]. Next, *M. luteus* may interact with other possibly more dangerous microorganisms of the skin, such as *Candida albicans* [12]. Finally, this microbe is also exposed to human humoral factors because it inhabits the same niches as other more studied counterparts. However, the concentration of 10^−6^ M (1.83 μg/mL) used in the work of Borrel et al. is potentially higher than that normally circulating in the bloodstream. The authors reasonably noted that the actual epinephrine concentration in sweat on skin has not been determined. Therefore, we decided to make our basis the epinephrine concentrations in blood plasma to get closer to natural environment of cutaneous strain *M. luteus* C01, testing a range of lower and higher concentrations to simulate potentially different concentrations of epinephrine in skin. According to Boyanova [28], the physiological (in normal conditions without stress and disorders) concentration of epinephrine in the plasma is up to 4.9 × 10-9 M (900 pg/mL). In some studies and models epinephrine effects were studied in parallel with norepinephrine – another catecholamine hormone, precursor of epinephrine with similar physiological role in humans(Bylund, 2015).

Recently, we published a short communication presenting the first data concerning the effects of epinephrine on *M. luteus,* where we observed a complex effect of 4.9 × 10-9 M of epinephrine on the growth of this microbe [29]. In this study, we expand our investigation and suggest the mechanisms underlying the effect of 4.9 × 10^−9^ M and higher concentrations of epinephrine on *M. luteus* C01. This study is the first in-depth investigation to use transcriptomics and other methods to study the effects of epinephrine on *M. luteus* biofilms.

## 2 Materials and methods

**Bacterial strains and growth conditions.***Micrococcus luteus* C01 was isolated from the skin of a healthy volunteer and identified by 16S rRNA sequencing, as described previously [30]. The bacterium was conserved at room temperature (RT) in glass tubes filled with 5 mL lysogeny broth (LB, Lennox, Dia-M, Moscow, Russia) with the addition of 0.3% agar (BD, USA), and cultures were grown on the surface of semisolid agar and covered with sterile mineral oil. For experiments examining biofilm growth, cultures were plated onto reinforced clostridial medium (RCM) with 1.5% agar, and single colonies were obtained. The RCM composition (g/L) was yeast extract 13, peptone– 10, sodium chloride – 5, sodium acetate– 3, glucose – 5, starch – 1, and L-cysteine-HCl – 0.5, pH 7.0. All reagents were obtained in Dia-M, Russia except L-cys-HCl (Biomerieux, France). The RCM medium was tested previously (data not shown) and demonstrated the optimal conditions for *M. luteus* C01 biofilm experiments with medium-level biofilm growth in comparison to lysogeny broth (LB) and tryptic soy broth (TSB) (not too abundant but significant biofilms growth to track the epinephrine effect). The RCM medium was sterilized in mild conditions at 112°C for 30 min to avoid sugar caramelization and destruction of other medium components? such asyeast extract. For each experiment, a single colony was inoculated in a 50 mL conical flask with 15 mL of LB and cultivated overnight at 33°C (close to skin physiological temperature [7]) and shaker speed 180 rpm. The resulting *M. luteus* cell suspension was adjusted with sterile physiological saline (PS, 0.9% NaCl in distilled water, pH 7.0) to a final OD_540 of_ 0.5 (3 × 10^7^ CFU/mL) for polytetrafluoroethylene (PTFE) cube experiments and confocal microscopy studies or 0.1 (6 × 10^6^ CFU/mL) for assessments of biofilms on glass fiber filters.

**Stock solutions of epinephrine.** Epinephrine (Sigma–Aldrich, USA) was dissolved in sterile Milli-Q water, and a series of stock solutions in sterile Milli-Q water were prepared for use in experiments. Under these conditions epinephrine is stable for a sufficiently long time [31]. Stock solutions were stored at −18 °C in 15 mL conical centrifuge tubes (ThermoFisher, USA), in the dark to avoid potential light-caused destruction of the hormone. Because of relatively unstable liquid homeostasis in skin (due to the alternation of sweating and drying periods), it is difficult to define the concentrations of epinephrine in *M. luteus* inhabiting microniches in skin, such as hair follicles and the stratum corneum [2]. Thus, we have taken 4.9 × 10^−9^ M (physiological) of epinephrine as the baseline concentration because, according to a recent review, it is close to the normal physiological concentration in blood plasma [28]. In experiments with PTFE cubes, the range of epinephrine concentrations (4.9 × 10^−12^, 4.9 × 10^−11^, 4.9 × 10^−10^, 2.4 × 10^−9^, 4.9 × 10^−9^, 9.8 × 10^−9^, 4.9 × 10^−7^, 4.9 × 10^−6^ M) was also tested. Higher concentrations of epinephrine were chosen to simulate different stress conditions when level of epinephrine in bloodstream increases or when there is a disorder in the human organism leading to a permanent increase in the epinephrine concentration [28], or potentially lower concentrations of the hormone which may be present in skin. Next, a concentration of 4.9 × 10^−9^ M was employed in the transcriptomic analysis, confocal microscopy studies and glass fiber filters assay × . All tubes, Petri plates and glass-bottom plates with biofilms of *M. luteus* C01 were also incubated in the dark when epinephrine was added. Due to the lack of information on the probable consumption of epinephrine by *M. luteus* C01 during its growth, and because of technical difficulties in measuring of epinephrine concentration in the small volume of the rich medium at each time point of an experiment, we did not inoculate any additional portions of epinephrine during the incubation. The hormone was inoculated once at the beginning of the experiment to make conditions standard.

**Biofilm growth on PTFE cubes.** Biofilms on cubes were grown as described in previous studies [30,32,33] with modifications. Briefly, in glass tubes of a standard volume 22 mL with screw plugs, 21 chemically pure cubes with a size of 4 × 4x4 mm were placed. After addition of 3 mL of the RCM to each tube, the tubes were capped loosely with screw plugs and autoclaved at 112 °C. After sterilization and cooling, appropriate epinephrine stock solutions were added to each tube to obtain different final epinephrine concentrations in the medium. Tubes without epinephrine addition were used as a positive control. Then, 50 μL of prepared cell suspension was added to each tube, and at least two tubes were used as negative controls without bacterial inoculation. The tubes were incubated at 33 °C at 180 rpm for 24 h or 72 h to obtain immature and mature biofilms, respectively [29]. After incubation, the OD_540_ was measured using of empty controls without bacterial inoculation, and biofilm CV or MTT staining was subsequently performed.

**Biofilm staining on PTFE cubes.** To analyze the total amount of biofilms on the PTFE surface, CV staining was used. Biofilms were stained as described previously [32]. Briefly, cubes were washed twice gently with room temperature tap water to remove the planktonic culture and fixed with 3 mL of 96% ethanol for 20 min. After fixation, ethanol was removed, cubes were dried, and 2 mL of the 0.5% CV solution in distilled water was added to each tube and incubated for 20 min at room temperature. Next, the CV was removed, and cubes were washed 6 times gently with RT tap water and placed into new clear glass tubes to be covered with 3 mL of 96% ethanol for dye extraction. The OD_590_ was measured after 30 min of extraction using of negative controls. OD measurements were performed using a spectrophotometer PE-5400VI (Ecroskhim, Russia).

To analyze the metabolic activity of biofilms on PTFE cubes, 3-(4,5-dimethyl-2-thiazolyl)-2,5-diphenyl-2H-tetrazolium bromide (MTT, Dia-M, Russia) was used [33]. Cubes were washed twice to remove planktonic suspension residues. After that step, 0.1% MTT solution in sterile LB medium was prepared, 3 mL of this solution was added to each tube, and biofilms were stained for 1 h at room temperature. Then, biofilms were washed three times gently with room temperature tap water, dried, and moved into new clear glass tubes, and 3 mL of dimethyl sulfoxide (DMSO, 99.9%, EKOS, Russia) was added to each tube for formazan extraction. Extraction was performed overnight in tubes sealed with Parafilm® (Amcor, Switzerland), and the OD_540_ was measured after extraction.

**DNAse I and Proteinase K succeptibility test.** Biofilms of *M. luteus* were grown on PTFE cubes for 24 h and 72 h in the presence of physiological concentrations of epinephrine and treated with proteinase K (Dia-M, Russia) and DNAse I (NEB, USA). Both enzymes were used in concentration 5 μg/mL. Proteinase K treatment was performed according to Ref. [34]. DNAse I treatment was conducted according to Ref. [35] and the manufacturer's protocol. Briefly, biofilms on cubes were washed twice with water and treated with an appropriate enzyme. Subsequently, biofilms were washed twice again, fixed in 96% ethanol and stained with CV as described before.

**Initial adhesion test and cell surface hydrophobicity in the presence of epinephrine**. Epinephrine can potentially change the initial adhesion of *M. luteus* C01 cells to PTFE. To examine the potential changes in initial cell adhesion in the presence of 4.9 × 10^−9^ M epinephrine, the potential decrease of in the OD_540_ of the *M. luteus* C01 cell suspension exposed to PTFE cubes in the system depicted above was measured. Briefly, a portion of the 24 h culture was diluted in the RCM with or without epinephrine up to an OD_540_ = 0.1. Three milliliters of the suspension was inoculated into chemically clean tubes with 21 PTFE cubes and incubated at 33°C at 150 rpm. The OD_540_ was measured at different time points (10, 20, 30 min) after the start of the experiment. Also, after 30 min of incubation cells were washed out from cubes and plated for CFU counts. Cells adherence to the cubes resulted in an OD decrease, and epinephrine could potentially alter this process. Additionally, cell surface hydrophobicity in the presence of epinephrine was measured as described by Gannesen et al., 2018 and [36] by the adhesion to hexadecane method.

**Biofilm growth on glass filters.** Glass microfiber filters (GMFF, Whatman® GF/F, USA) were used as carriers for biofilms of *M. luteus* to analyze the metabolic activity and colony forming unit (CFU) amounts. Biofilms were studied as described previously [37]. RCM with 1.5% agar was melted and stored in a 55°C water bath for the experiment. An appropriate volume of the epinephrine stock solution was dropped into a sterile 100 mL glass vial. Next, 20 mL of RCM-agar was addedto the vial, mixed properly for 5 s and plated onto a 90 mm Petri dish. RCM-agar without epinephrine was used as a control. After medium solidification, six sterile Ø 21 mm GMFFs were placed on the agar surface, and 20 μL of *M. luteus* suspension with OD_540_ 0.1 was dropped on the center of each filter. A filter without inoculation was used as a negative control for MTT staining. Biofilms were grown for 24 h and 72 h, after which 3 filters were stained with MTT. Briefly, the filters wereplaced in a clean 6-well plate, with one filter per well, after addition 3 mL of LB containing 0.1% MTT (mass/volume percent) to each well and resting for 30 min at room temperature. Then, the filters were gently washed with distilled water to remove the resting MTT solution and stopthe reaction, placed into another 6-well plate and covered with 3 mL of DMSO per filter to extract the formazan. The remaining 3 filters from a plate were dispersed for CFU counts. Briefly, each filter was placed into a glass tube filled with 10 mL of PS, dispersed with a glass stick and vortexed for 1 min at medium speed. A series of 10X dilutions was made from the resulting suspension and then 20 μL of a final suspension was plated onto a Petri dish. Additionally, 10 μL of each nondiluted filter suspension was fixed and stained with CV for cell aggregation analysis using light microscopy (Carl Zeiss Jena, Germany) analysis at 900x magnification with immersion oil (Merck, Germany). The samples were visually evaluated in 50–100 fields. For each sample, at least 5 of the most representative photos were taken (microscope-attached camera ToupView, China), and single cells, cell aggregates and aggregate ratios were calculated manually on photos. Additionally, the change in the amount of aggregates in the presence of the hormone was analyzed..

**Confocal laser scanning microscopy (CLSM).** CLSM was performed as described previously [38]. Briefly, biofilms were grown in 24-well black plates with flat glass bottoms (Eppendorf, Germany). One mLmilliliter per well of RCM with or without 4.9 × 10^−9^ M epinephrine was added to a plate, and 17 μL of prepared M. *luteus* cell suspension with OD_540_ 0.5 was added to each well. The plates were incubated for 24 and 72 h at 33°C at a shaker speed of 180 rpm to obtain well-established biofilms on the flat glass surface. After incubation, the biofilms in wells were washed with sterile PS to remove unattached cells. Then, the samples were stained with SYTO 9 Green dye (Molecular Probes (Thermo Fisher Scientific)): the manufacturer-produced dye solution in DMSO was diluted 1000 times in PS, and 200 μL of the obtained solution was applied to the wells. Staining was performed for 20 min at room temperature in the dark, whereupon the liquid was discarded and the wells were washed two times with PS. ProLong Gold Antifade Mountant liquid (1–2 drops, Molecular Probes (Thermo Fisher Scientific)) was added, and plates were incubated overnight at 4°С. The samples were analyzed with an LSM 510 Meta inverted confocal microscope (Carl Zeiss, Germany) at an argon laser wavelength of 488 nm with a 63 × /1.2 water immersion lens. To limit the spectral range of fluorescence, a longpass filter with a transmission above 505 nm was used. The optical resolution of the system in routine measurements is 0.3 μm along the focal plane and 0.7 μm along the optical axis of the lens. The pixel size of the digitized image is 0.12 × 0.12 μm, the size of the area is 146.4 × 146.4 μ m, and the scanning step along the z axis is 1 μm. While obtaining more detailed 3-D images, the confocal aperture was close to a size corresponding to 0.7 Airy disk, and the z-scan was reduced to 0.5 μm. The microscopic studies were conducted, and the sample data files were obtained using Carl Zeiss LSM 510 Software, Version 3.2 (Carl Zeiss, Germany). The data obtained were processed using the ImageJ package in the Comstat2 plug-in software (based on predesigned computational algorithms). For each well, at least 5 3D-photos were taken for quantitative analysis. Four parameters were determined: biofilm average thickness (μm); average biomass distribution per area unit (ABD, μm^3^/μm^2^); and biofilm surface square (BSS, μm^2^) Images of 3-dimensional biofilm structure were obtained using the Zen 2.3 Blue Edition (Carl Zeiss microscopy GmbH). Experiments were conducted in triplicate.

**Total RNA isolation from *M. luteus* biofilms.** Before RNA isolation, crushed glass for cell disruption was prepared of typical filament lamp glass: four lamps were broken in the kettle and the glass was ground up, washed to chemical purity with bichrome solution, treated with 3% H_2_O_2_ to avoid any residual RNAse activity and sterilized by autoclaving. All vessels, pestles and nonmetal instruments were prepared chemically pure, pretreated with H_2_O_2_ and autoclaved similarly to crushed glass. The electrophoresis cell, gel combs and plates were also pretreated with H_2_O_2_ solution.

Petri dishes with RCM-agar with and without the addition of 4.9 × 10^−9^ M epinephrine were prepared as described above. Two sterile Ø 21 mm GMFFs (one filter was in reserve) were placed onto the agar surface per dish. Beforehand, the prepared *M. luteus* cell suspension with an OD_540_ 0.5 was diluted 100 times with sterile PS to a final CFU count of 3 × 10^5^ CFU/mL, and 25 μL of this suspension was inoculated on the center of each filter. Biofilms were grown for 24 h. A Qiagen RNeasy® Mini Kit (Qiagen, Germany) was used for total RNA extraction. The manufacturer's protocol was performed with changes. After incubation, the filter with the biomass was placed into the porcelain mortar. Twenty-five microlitersof RLT buffer (with addition of mercaptoethanol according to the manufacturer's protocol) was applied onto the biofilm, and 0.5 cm^3^ of crushed glass was placed onto the filter. The mortar was filled top to bottom with liquid N_2_ [39], and the pestle was also cooled in liquid N_2_. When 3/4 of the N_2_ volume was evaporated, the mass in the mortar was vigorously smashed using the pestle until N_2_ was completely gone and before the moment of ice melting. The cycle with N_2_ addition and smashing was then repeated 4 times. Ultimately, 1 mL of RLT buffer was added to the ice-cold mortar with the resulting frozen homogenous powder of glass with disrupted cells, mL, and the mass was vigorously mixed until the ice was melted. mLThe suspension was then transferred into a sterile 2 mL Eppendorf tube, and the glass powder was pelleted in an Eppendorf Minispin centrifuge (Germany) at 13000 rpm (11700 *g*) and room temperature for 15 s. The supernatant was transferred into RNeasy columns, and all subsequent manipulations were conducted according to the manufacturer's protocol.

Agarose gel electrophoresis was performed to check the quality of the total RNA extracted from biofilms. A 1% agarose (Sigma, USA) gel was used, and 0.01% v/v ethidium bromide (Sigma, USA) was inoculated into the gel. For each experiment, fresh 1X TAE buffer was prepared to fill the electrophoresis cell. The RNA samples were separated at 65 V for 70 min. Ribosomal RNA bands were the main marker of successful extraction and other RNAs were visualized using of Bio–Rad Gel Doc XR System w/Universal Hood II (Bio–Rad, USA) and Gel Doc XR software. Samples of RNA were stored at −80 °C. Experiments were conducted in duplicate.

**Total RNA sequencing.** The concentration of RNA in the samples was measured with Qubit 2.0 (Invitrogen, USA). Ribosomal RNA depletion was conducted using the Illumina Ribo-Zero Plus rRNA Depletion Kit (Illumina, USA). Depletion was performed according to the manufacturer's protocol with 120 ng of total RNA for each sample. Next, RNA libraries were prepared using the NEBNext Ultra™ II Directional RNA Library Prep Kit for Illumina (New England Biolabs® Inc., USA) according to the manufacturer's protocol. RNA was fragmented for 5 min. Libraries were indexed using of the index primers set NEBNext Multiplex Oligos for Illumina (Dual Index Primers Set 2) from New England Biolabs® Inc., USA. Library amplification was carried out in 15 PCR cycles. Sequencing was conducted in single-end mode in three runs: the first on a HiSeq 4000 with read length of 151 base pairs (bp), the second on MiSeq with a read length of 301 bp and the third on HiSeq 4000 with read length of 51 bp. Reads were generated by bcl2fastq 2.20 [40]without allowing mismatches in sequencing indexes (“--barcode-mismatches = 0″).

**Read preprocessing and quality control.** Reads were preprocessed by Trimmomatic 0.39 [41] performing the following procedures consecutively:

1. Adapter trimming.
2. Removal of bases with a quality below 3 from the 3′ ends of reads.
3. Removal of 3′ ends of reads starting with 4 bp-long regions with an average quality below 15 (option “SLIDINGWINDOW:4:15″).
4. Removal of reads with average quality below 20.
5. Removal of reads shorter than 30 bp.

To analyze the level of contamination in reads, 1000 reads from each library were aligned by BLASTN 2.9.0 [42] to the NCBI nt database with a maximum e-value of 10^−5^. The taxonomy of the best BLAST hit according to the NCBI Taxonomy database was used to infer the taxonomy of the read source. The NCBI nt and NCBI Taxonomy databases were current as of 24 April 2020. The analysis showed that the level of contamination is negligible (Table S1.)

**Differential expression analysis.** To analyze differential expression, reads were aligned to the genome of *M. luteus* NCTC 2665 (NCBI accession NC_012803.1) by BWA 0.7.17 [43] using the BWA-MEM algorithm. The numbers of reads belonging to different genes were calculated by Salmon 1.3.0 [44] with 20 Gibbs samples and a correction for GC bias. Then, the differential expression was analyzed by DeSeq2 1.22.2 with the default parameters. Hierarchical clustering was performed using the hclust function of the R programming language with the complete linkage clustering algorithm. Principal component analysis was performed using the plotPCA function of DeSeq2.

**Quantitative RT**–**PCR.** To confirm, the results of differential expression analysis. RT–PCR was conducted. Newly extracted total RNA samples were obtained from three independent experiments as described above. First strand complementary DNA (cDNA) synthesis for real-time qPCR was performed using Moloney Mouse Leukemia Virus reverse transcriptase according to the manufacturer's protocol (Evrogen, Russia). Specific primers (Supplementary data Table S3) were applied for the synthesis of unique cDNA fragments. At least three pairs of primers for each gene with differential expression were selected using Unipro UGENE v.38.1 (Okonechnikov et al., 2012) with the built-in Primer3 module and the primer selection function for RT-PCR. The primers were checked *in silico* using the web resource insilico [45]. The annotated *M. luteus* genome NTCT 2665 was used as a reference. To find the optimal primer pairs, hybridization with total DNA of *M. luteus* C01 was performed once before the RT–PCR experiments. Total DNA was extracted from 24 h suspension cultures of *M. luteus* C01 using of Wizard® Genomic DNA Purification Kit (Promega, USA). For improved cell wall disruption, the pellet was previously frozen with liquid nitrogen and milled with glass as described for the total RNA extraction protocol.

RT-PCR was performed in PB PCR buffer (Syntol, Russia) in the presence of SYBR Green I and the passive reference dye ROX for fluorescent signal normalization. For each sample, detection was conducted twice. ddH2O (Syntol, Russia) was used as a negative control. Amplification was carried out with the CFX96 Touch™ RT–PCR detection system (Bio-Rad, USA) in the following reaction regime: polymerase activation for 5 min at 95°C followed by 40 cycles of 15 s at 95 °C–20 s at 55 °C–40 s at 62 °C. The differential expression of selected genes was measured in comparison to a control nonprocessed sample. Average means of target genes were normalized in comparison with the reference gene MLUT_08120 (F0 subunit of the conserved ATP synthase). The amount of a target normalized to an endogenic control and a calibrator was determined using the Ct (ΔΔСt) comparison method with formula 2^−ΔΔ^ ^Ct^. Data analysis was performed using CFX ManangerTM Software v. 1.6.

***In silico* protein sequence analysis.** Protein homologs searches, alignment analyses, and building similarity trees were performed using NCBI BLAST tools [46] (https://blast.ncbi.nlm.nih.gov/Blast.cgi), the NCBI protein database [47] (https://www.ncbi.nlm.nih.gov/protein) and the UniProt database [48].

**Microbiological statistics.** All experiments were conducted at least in triplicate. RNA extraction was performed in duplicate. Statistical analysis of the data (except the RNA sequencing described above) was performed using the nonparametric Mann–Whitney *U* test. Analysis of the CFU counts was performed using the nonparametric Wilcoxon Z-test. q-values were calculated from p-value in each experiment using false discovery rate correction, as proposed previously [49]. q-values are indicated on the data plots.

All microbiological data plots were designed using Microsoft EXCEL 2007 Software. Average relative values (the control without addition of epinephrine was designated as 100%) were plotted on the graphs, and the standard error of the mean was depicted as error bars.

## 3 Results

**Effect of epinephrine on *M. luteus* biofilms on PTFE cubes.** × mLIn this study, we employed CV staining to further test the effect of eight concentrations of epinephrine, which were decreased and increased in 10X increments and 2X and 0.5X physiological concentrations, after 72 h of incubation to determine any correlation between the concentration of epinephrine and its effect on biofilm growth. An example of CV stained cubes is depicted in the Supplementary material, Fig. S1A. Also, we tested physiological concentrations to confirm the data obtained by Danilova and co-authors [29]. We found that at physiological concentrations (4.9 × 10^−9^ M) biofilms stained with CV accounted for 157.2 ± 24.7% of the control biofilms (Fig. 1, supplementary data, Table S1). With a 2-fold increase in concentration (Fig. 1), the stimulatory effect of epinephrine vanished, and at 4.9 × 10^−8^ M, 4.9 × 10^−7^ M and 4.9 × 10^−6^ M in RCM, this hormone had no effect on the growth of *M. luteus*. In the case of decreased concentrations there was no significant effect at any concentration. Furthermore, no effect of epinephrine on planktonic growth was observed at any concentration. This finding provides direct evidence that epinephrine i) acts in a biofilm-specific manner and ii) has a concentration-dependent effect, and this phenomenon is difficult to explainbased on the relationship between an effect and an increase or decrease in concentration. These results demonstrate that epinephrine has a pronounced stimulatory effect on the total biomass of mature 72-h *M. luteus* C01 biofilms only at physiological concentrations (4.9 × 10^−9^ M).

**Fig. 1:**
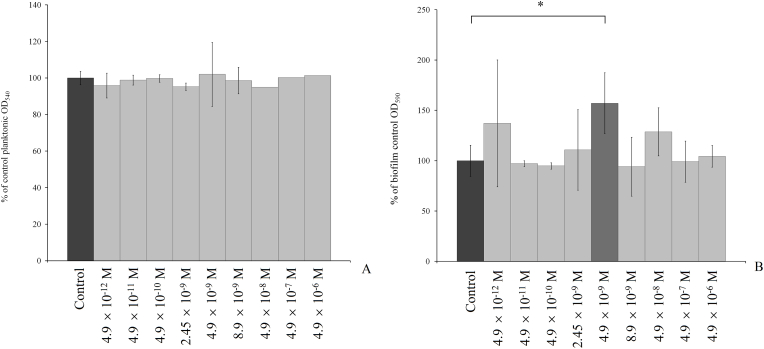
Effects of different concentrations of epinephrine on the growth of 72 h *M. luteus* C01 planktonic cultures (A) and biofilms (B) on PTFE cubes compared with the control without additions.. The absence of an asterisks means q > 0.05, and * means q ˂ 0.05.

Based on these findings, we examined the physiological concentration in further studies. Specifically, we tested the impact of 4.9 × 10^−9^ M epinephrine on immature 24 h biofilms of *M. luteus* C01 by CV and MTT staining, as was described by Danilova et al. on PTFE cubes [29]. Comparison of two different staining methods allowed us to predict the potential manner of epinephrine action regarding whether it affects cell growth or matrix synthesis in *M. luteus* biofilms. In this experiment, we found that in contrast to mature 72-h biofilms, there was a significant decrease in total biofilm biomass stained with CV (74.4 ± 9.1%) in comparison to the control (Fig. 2B). In parallel, metabolic activity measurements showed no effect of epinephrine. Additionally, we tested the 4.9 × 10^−12^ M epinephrine concentration and found no significant effect on biofilms stained with CV, however metabolic activity was insignificantly altered according to the Mann–Whitney test (104.7 ± 25.2% of control, Fig. 2C). We also tested MTT staining on 72-h biofilms in the presence of both concentrations of epinephrine and found no effect of epinephrine biofilms: 98.9 ± 16.3% and 109.0 ± 18.4% at physiological and 1/1000X physiological concentrations respectively, which were not statistically significant (Fig. 2C). Taking into account these data and the results of a previous study, we proposed that epinephrine could affect either matrix synthesis (especially in mature biofilms) or the ratio of certain metabolic pathways that do not lead to MTT reduction.

**Fig. 2:**
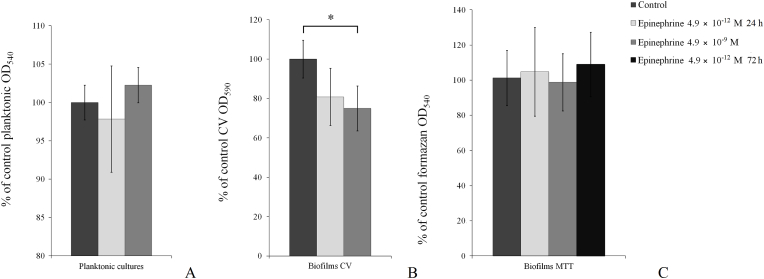
Effect of 4.9 × 10^−9^ M epinephrine on 24 h and 72 h planktonic cultures and biofilms of *M. luteus* C01 on PTFE cubes. A- planktonic cultures; B – biofilms stained with CV; C – biofilms stained with MTT. mLThe absence of an asterisks means q > 0.05,. * means q ˂ 0.05..

### 3.1 Enzymatic treatment of *M. luteus* C01 biofilms and adhesion properties

In these experiments, it was demonstrated that proteinase K had no effect on *M. luteus* C01 24 h and 72 h preformed biofilms, which suggested a reduced impact of proteins in the matrix composition. DNAse I had no effect on 24 h immature biofilms, but it reduced the stimulatory effect of epinephrine on 72 h mature biofilms (Fig. 3). These results suggested that extracellular DNA had greater impact on matrix composition in the presence of 4.9 × 10^−9^ M epinephrine, and that epinephrine potentially stimulates matrix synthesis in biofilms.

**Fig. 3:**
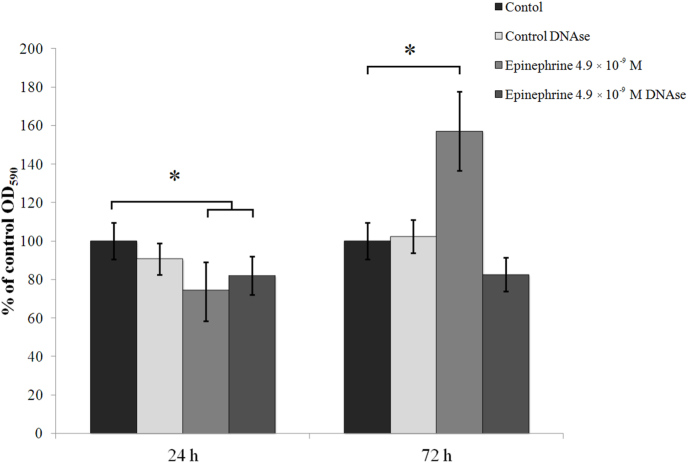
Effect of DNAse I on *M. luteus* C01 biofilms on PTFE cubes. 24-h and 72-h biofilms were treated with the enzyme for 30 min at 37°C.. The absence of asterisks means q > 0.05, and * means q ˂ 0.05.

This phenomenon was confirmed by data obtained using the microbial adhesion to hexadecane method and adhesion tests. No effect was found during the initial adhesion (data not shown). In the hexadecane method, in the control and in the presence of epinephrine the aqueous phase OD_400_ was 58.2 ± 12.6% and 54.5 ± 13.2%. Thus, the mechanism of epinephrine action was not based on cellular surface property changes, but on switching of other metabolic pathways and matrix synthesis modifications.

**Fig. 4:**
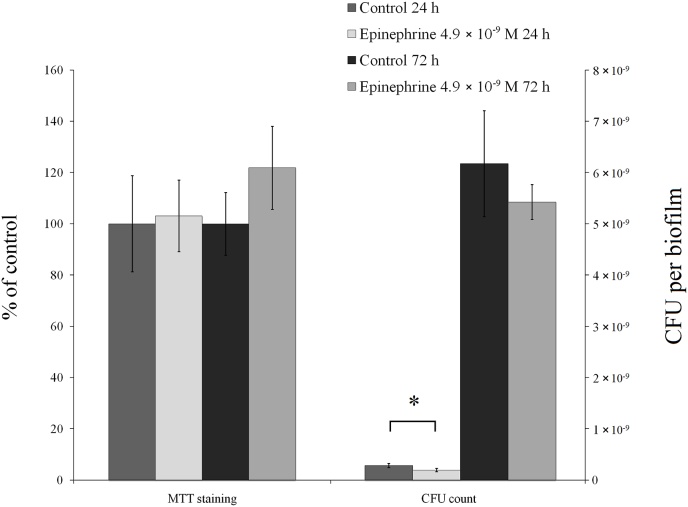
Effect of 4.9 × 10^−9^ M epinephrine on 24 h and 72 h biofilms of *M. luteus* C01 on GMFFs. Biofilms were stained with MTT for 30 min at RT and in parallel disrupted for CFU count. The absence of an asterisks means q > 0.05, and * means q ˂ 0.05.

**Effect of epinephrine on biofilms on glass fiber filters.** Due to the microfiber structure of glass microfiber filters (GMFFs), they may represent a kind of model of parenchymatous skin derma structure [50,51]. Hence, the use of GMFFs as a biofilm carrier allows the simulation of biofilms growing in skin wounds and lesions. Examples of *M. luteus* C01 biofilms on the GMFF are depicted in the Supplementary material, Fig. S1B. In a previous study, epinephrine had no effect on the CFU amount and stimulated metabolic activity in mature 72-h *M. luteus* C01 biofilms by 25.3% [29]. In this study, we investigated both 24-h and 72-h biofilms in the presence of 4.9 × 10^−9^ M epinephrine (Fig. 4). The data obtained for 72-h biofilms were consistent previously published results(– [29]: a small statistically insignificant tendency to inhibit the CFU counts and a tendency to stimulate MTT staining (121.9 ± 28.4% of control, Fig. 4, Supplementary data Table S2). In 24-h biofilms, a significant decrease in the CFU amount (68.1 ± 4.3% of control) was snown in the presence of the hormone. In control samples the average amount of CFU was 2.97 × 10^9^ ± 3.9 × 10^8^ in the biofilm. In the presence of epinephrine the number of CFUs decreased to 2.02 × 10^9^ ± 4.4 × 10^7^. Additionally, no effect of epinephrine was detected on metabolic activity in 24 h biofilms on the GMFFs, which was not consistent with the findings obtained on PTFE cubes. This discrepancy can be explained by differences in the model systems. On PTFE cubes, biofilms form in the presence of planktonic culture: most cells grow in the liquid phase, and a small portion of the cells adhere to the PTFE surface and start to form biofilms. On the GMFFs on an agar surface, cell adhesion occurs virtually immediately after inoculation because of the lack of free liquid in a sufficient volume to form planktonic culture. Thus, on the GMFF surface, the initial adhesion stage occurs instantaneously and different genes may be expressed differently in comparison to the PTFE cube system.

**Fig. 5:**
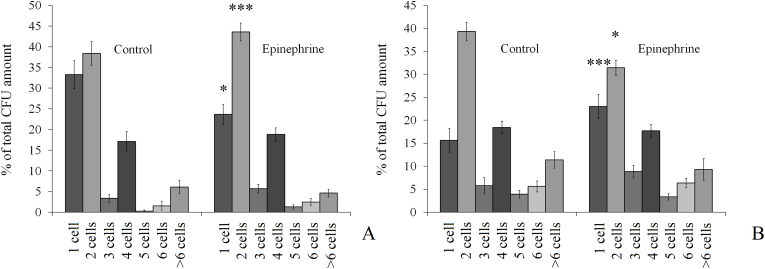
The frequency of cell aggregates of different sizes and single *M. luteus* C01 cells as a percentage of the total CFU amount analyzed with light microscopy. A – aggregation in 24-h biofilms; B – aggregation in 72-h biofilms. The absence of asterisks means q > 0.05, and * means q ˂ 0.05.

We used light microscopy of CV-stained samples to control the aggregation of cells after disruption of the filters. We analyzed cell aggregation after both 24 h and 72 h of incubation. Aggregate size was evaluated on glass slides stained with CV. Representative photos of the samples are presented in the supplementary data, Fig. S2. We found that after 24 h of incubation in control samples 33.2 ± 3.4% of CFUs were potentially formed from single cells, and 38.4 ± 2.9% were formed from two-cell aggregates (Fig. 5A). The third largest CFU group was those that started to grow from cell tetrads (17.1 ± 2.4%). In the presence of 4.9 × 10^−9^ M epinephrine, the single-cell CFU amount was reduced to 23.6 ± 2.4%, and the cell pair CFUs increased up to 43.6 ± 2.1%. Other cell aggregates were not significantly affected. The number of larger (˃ 6 cells) aggregates increased slightly from 1.5 ± 1.1% in controls to 2.5 ± 0.8% in thepresence of the hormone. The aggregation percentage increased from 66.7 ± 7.6 % in control to 76.3 ± 5.5% in the presence of the hormone (q ˂ 0.05). Hence, epinephrine increased the number of cell pairs in immature 24-h biofilms. In mature 72-h biofilms in control samples the single-cell CFU amount was 15.6 ± 2.6 %, cell pair CFU amount was 39.3 ± 2.0%, cell tetrade CFU amount was about 18.4 ± 1.3% (Fig. 5B). In the presence of epinephrine single cells formed 23.0 ± 2.6%, the of CFU, cell pairs – 31.4 ± 1.7%, and the cell tetrad amount and other aggregates were not changed significantly. The aggregation ratio in the presence of the hormone decreased after 72 h of incubation from 84.5 ± 2.5% in control to 78.4 ± 2.6%. Thus, in immature and mature biofilms epinephrine had opposite effects, especially on the balance of single cells – cell pairs. Since *M. luteus* normally grows in cell packs and tetrads, epinephrine can potentially change the processes of cell pair and tetrad formation.

Hence, on the one hand, there was a decrease in the CFU amount in combination with an increased MTT reduction (i.e., metabolic activity of cells) in immature biofilms on PTFE cubes. On the other hand, there was a decrease in CFU counts in biofilms on GMFFs in conjunction with no effect on metabolic activity and an increased number of cell pairs forming CFUs. This can potentially be explained by changes in the cell pair division process in the presence of epinephrine.

**Fig. 6:**
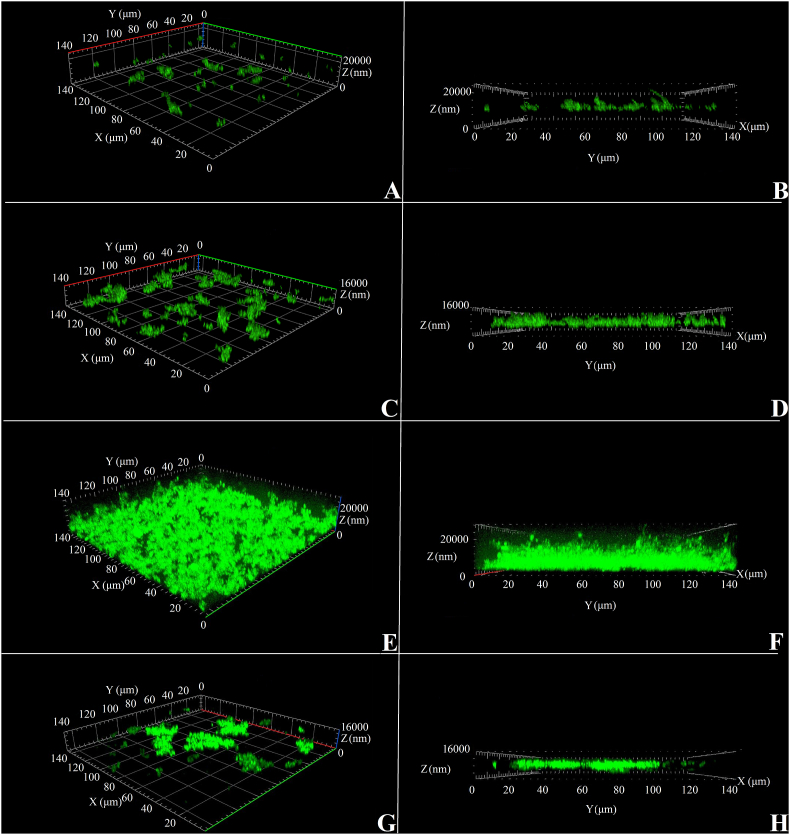
CLSM 3D images of *M. luteus* C01 biofilms stained with SYTO 9 Green. (A, B) – general (A) and side (B) views of a 24 h control sample; (C, D) – general (C) and side (D) views of a 24 h sample with added epinephrine (4.9 × 10^−9^ M); (E, F) – general (E) and side (F) views of a 72 h control sample; (G, H) – general (G) and side (H) views of a 72 h sample with added epinephrine (4.9 × 10^−9^ M). (For interpretation of the references to colour in this figure legend, the reader is referred to the Web version of this article.)

**Fig. 7:**
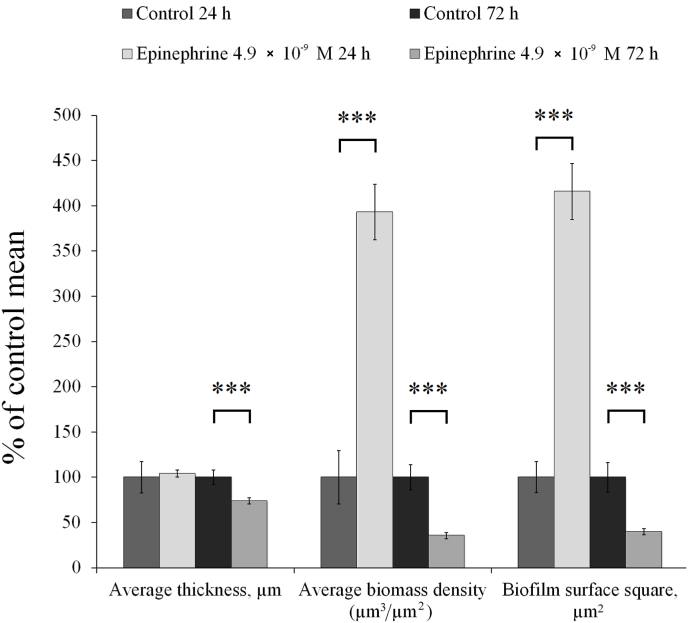
Parameters of *M. luteus* C01 biofilms analyzed by CLSM. The absence of asterisks means q > 0.05, and *** means q ˂ 0.001.

**Confocal laser scanning microscopy (CLSM) of biofilms.** A CLSM study of the architecture of immature (24 h) and mature (72 h) *M. luteus* C01 biofilms in the presence of epinephrine demonstrated a decrease in biofilm thickness and volume in the presence of the hormone (Fig. 6 and 7). Biofilms were stained with SYTO 9 Green, a nonspecific DNA-binding dye. Due to this SYTO 9 feature, only the cell biomass in a biofilm can be detected, and a potentially superior biomass of extracellular matrix may be overlooked because of the low extracellular DNA amount and general penetrability of the matrix. Biofilms in 72-h control samples looked like a well-established multilayer cell mate surrounded by matrix with a weak green glowing (Fig. 6E and F), while after 24 h biofilms looked like separate microcolonies (Fig. 6A,C).

After 24 h biofilms of *M. luteus* C01 were actually clusters of microcolonies without a continuous surface. Interestingly, in the presence of 4.9 × 10^−9^ M epinephrine, microcolonies (Fig. 6C and D, Fig. 7) were larger than in the control (Fig, 6A and B), and their average biomass distribution per area unit (ABD) was only 393.5 ± 30.7% of the control ABD (0.3 ± 0.1 μm^3^/μm^2^ in the control and 1.1 ± 0.1 μm^3^/μm^2^ with epinephrine). Additionally, the biofilm surface square (BSS) in the presence of the hormone was 416.1 ± 31.3% of the average control BSS (2.4 × 10^4^ ± 4.0 × 10^3^ μm^2^ in control and 9.8 × 10^4^ ± 7.3 × 10^3^ μm^2^ with epinephrine). The last parameter suggests that in addition to less volume, microcolonies in biofilms became bulkier with 3D-organization. In parallel, the average thickness in the presence of the hormone was not significantly increased. In mature biofilms, the opposite effect of epinephrine was demonstrated: biofilms were shown to be more fragmentary and to exhibit considerably weaker growth than in control samples (Fig. 6E and F). All parameters of the biofilms were significantly changed (Fig. 7): biofilms were 26.2 ± 3.0% thinner than in the control (16.2 ± 1.3 μm in the control and 11.9 ± 0.5 μm with epinephrine respectively), and their ABD was only 35.6 ± 3.4% of the control ABD (8.6 ± 1.2 μm^3^/μm^2^ in control and 3.1 ± 0.3 μm^3^/μm^2^ with epinephrine respectively). Additionally, BSS in the presence of the hormone was 40.5 ± 3.6% of the average control BSS (8.7 × 10^5^ ± 1.4 × 10^5^ μm^2^ in the control and3.5 × 10^5^ ± 3.1 × 10^4^ μm^2^ with epinephrine, respectively). The biofilms became more laminar, more bladed and less 3D-organized (Fig. 5G and H).

The difference in effect on 24-h and 72-h biofilms may be explained by the changes in epinephrine stimulation of *M. luteus* C01 microcolony formation on the glass in immature biofilms, which correlates with an increase in cell pairs in GMFFs. Taking into account the CV staining on PTFE cubes, one might speculate that the occurrence of a switch between matrix synthesis and microcolony formation: the changed matrix synthesis and composition is indicative of less stable microcolonies on PTFE in mature biofilms. In immature biofilms, changes in the cell division process and in matrix synthesis potentially lead to an increase in the size of microcolonies.

**RNA-seq analysis and quantitative RT–PCR.** Agarose gel electrophoresis showed a good quality of total RNA extracted from biofilm biomass. Principal component analysis (PCA) and hierarchical clustering used as methods of quality control suggested similarity between replicates in RNA-seq (Supplementary data, Fig. S3-A and S3-B). Additionally, the analysis of contamination revealed a minimal presence of contaminant RNA (Supplementary data, Table S4). We assessed wether the gene expression change was significant when the fold change was at least 1 or -1 (100% upregulation or 50% downregulation), withq ˂ 0.05. According to 16S rRNA sequencing, *M. luteus* strain NCTC 2665 was the closest to our strain *M. luteus* C01 strain. Therefore, we decided to align the full genome of the NCTC 2665 strain. The results obtained using RNA-seq and subsequent RT-PCR demonstrated that epinephrine at physiological concentrations changed the expression of 7 genes (Table 1.). Four genes demonstrated decreased expression levels, with the first being SDR-family oxidoreductase MLMLMLML.

**Table 1:**
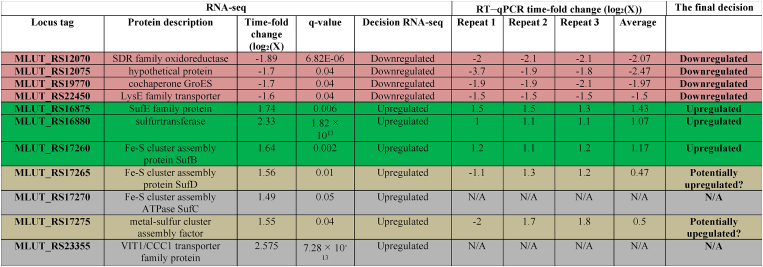
RNA-seq and RT–qPCR analysis of *M. luteus* C01 in the presence of 4.9 × 10^−9^ M epinephrine. RNA-seq was conducted in duplicate, and RT–qPCR was conducted in triplicate.

The second was the gene MLUT_RS12075 of a hypothetical protein (WP_010079694.1 in the NCBI protein database). This gene encodes 476 amino acids and two conserved EAL domains, which enabled us to propose its affiliation with the group of c-di-GMP phosphodiesterases, as described previously [52,53]. ML.

The third and fourth downregulated genes were MLUT_RS19770 of cochaperone GroES and MLUT_RS22450, encoding the LysE family transporter,. This gene encodes an L-lysine exporter with 29.1% (e value 2 × 10^−23^) similarity to the one described for *Corynebacterium glutamicum* [54] and mentioned in the accession description. Lysine is an important compound for biofilm formation and surface colonization [55]. Also it was previously shown that lysine can inhibit biofilm growth in *E. coli* [56]. Moreover, lysine can strengthen the inhibitory effects of other toxic molecules, such as antibiotics [57,58].

Three genes were upregulated in the presence of epinephrine, and two of them encode proteins directly involved in Fe–S cluster assembly processes and belong to the Suf system. MLUT_RS16875 encodes SufE (log_2_(fold change) = 1.741 ± 0.177) protein – an acceptor of sulfur from SufS through SufB, MLUT_RS17260 encodes SufB proteinMLML The SufBCD complex stimulates the synthesis of Fe–S clusters. ML.

The last upregulated gene was MLUT_RS16880 of sulfurtransferase ML. The upregulated sulfurtransferase gene is one of the four genes in *M. luteus* encoding sulfurtransferases, and its product catalyzes the production of thiocyanate. Expression of the other three genes was not affected by epinephrine. The MLUT_RS20535 product catalyzes oxygen-dependent 5-hydroxyuridine (ho5U) modification at position 34 in tRNAs; the MLUT_RS22835 product TusA participates in redox regulation; the MLUT_RS15435 product, similar to upregulated MLUT_RS16880, catalyzes detoxification of hydrogen cyanide by thiocyanate production. None of the three unaffected genes were found to contain EAL, GGDEF or HD-GYP domains.

MLMLMLML × There were also four genes for which the obtained results using RNA-seq and RT-PCR were controversial. These genes are MLUT_RS23355, MLUT_RS17265, MLUT_RS17270 and MLUT_RS17275 (Table 1). Hence, we avoided any decision on these genes. MLMLMLMLMLMLML

## 4 Discussion

The effects of epinephrine on the human microbiota have been studied for a long time.However, despite the considerable advances made in the last two decades, more studies must be conducted on the interactions between the very complex microbial community of skin and human organisms. Based on our data and data accumulated in recent decades, we can suggest with confidence that every humoral regulator affects the microbiota under certain conditions. Another question concerns to what extent conditionsreconstituted in laboratories enable an adequate analysis of the skin microbiota. We have not determined that in the natural microenvironment of *M. luteus* in human skin, the studied concentration of epinephrine or any other hormone is similar to that in blood plasma [18], but we also cannot deny its potential presence and, hence, effects.

*M. luteus* is a bacterium that has not been researched in deptht, probably because of its relative safety for human health, since host-microbiota interactions are now mostly of medical interest. Nonetheless, *M. luteus* is an important part of the cutaneous community [2,3], and neglect of its behavior is rather short-sighted, since this bacterium can affect other members of the community. Additionally, behavioral changes in *M. luteus* can potentially lead to skin homeostasis shifts. As we demonstrated using PTFE cubes and as it was demonstrated previously the effect of epinephrine (and at least some other hormones) is dose-dependent [7,37,59]^,^. This property could provide an additional reason for skin disorders during stress conditions and increased epinephrine production, such as acne vulgaris under psychological stress conditions [60].^.^It is likely that potential changes in *M. luteus* behavior in skin in the presence of high epinephrine concentrations shift the microbiota homeostasis and (directly or indirectly) affect acne development. Further studies are needed to elucidate the interactions between *M. luteus* and other cutaneous bacteria under both normal and stress conditions, as well as to determine the role of micrococci in acne development.

It is important to notice first that epinephrine affects mostly biofilms and not planktonic cultures of *M. luteus*, which issimilar to the manner of action of natriuretic peptides [7,37], potentially allowing us to suggest a relationship between biofilms as a primary bacterial lifeform in skin, and human humoral regulatory systems. As human skin normally does not allow bacteria to form suspension cultures due to the lack of a free liquid in an appropriate volume, microorganisms must attach to skin cells, to sebum exudates and to each other to form biofilms as shown previously (Jahns et al., 2014). It has been previously shown that epinephrine affects processes in many *P. aeruginosa* that are mostly associated with biofilm formation [61]. In *E. coli* deletion in QseC (sensor protein, member of a two-component regulatory system QseB/QseC) leads to a significant biofilm growth decrease [62]. Based on these facts we suggest here that hormones are potentially closely related to the regulation of biofilms of human microbiota.

Another interesting aspect is that epinephrine seemed to be active at concentrations close to those present in blood plasma and when concentration increased the effect seemed to disappear at least on PTFE cubes. Based on the previously obtained data for estradiol concentrations in the endometrium [63,64], in human skin, the concentration of epinephrine may be suggested as lower than in the bloodstream. Thus. we can propose that epinephrine affects *M. luteus* biofilms under stress conditions. It correlates with data of Borrel and colleagues, which shown the impact of epinephrine on *C. acnes* in high concentration, and it allows us to suggest the global epinephrine-mediated regulation of the human skin microbiota under stress conditions. Howerer. the absence of effect of epinephrine in higher concentrations may be explained by potential change of epinephrine targets in cells which switches its effect making biofilm growth “unchanged” in comparison with control. A potential biofilm assembly/disassembly balance due to epinephrine involvement in QS-like intercellular signal systems could also be a reason. These hypotheses are speculative and require approval or rejection in future studies.

We must stipulate that the relationship between the gene expression level and amount of a final translational product is complex, and it is necessary to take into consideration the possibility of discrepancies between real and predicted situationsNevertheless, the effect of epinephrine seems to be complex and multidirectional. On the one hand, this hormone potentially stimulates a number of processes in biofilms. The partial expression of Fe–S cluster assembly *sufBCDE* genes may explain the increased MTT reduction ratio. NAD(H) oxidoreductases also contain Fe–S clusters [65], and there may be a link between increased an MTT reduction ratio [66] and increased Fe–S cluster assembly expression.

In parallel with hypothetical matrix stimulation, epinephrine was observed to decrease the amount of cell biomass in *M. luteus* C01 biofilms in different model systems (GMFFs on Petri dishes, CLSM glass bottom plates) and changes in the amounts of single cells and cell pairs in suspension. While after 24 h of incubation *M. luteus* CFU single cell amounts were reduced in the presence of epinephrine in favor of cell pairs, in mature 72-h biofilms an opposite effect of the hormone was observed. We suggest that in these instances, there can potentially, on the one hand, be indirect lysine-mediated inhibition in immature biofilms based on the ability of this hormone to enhance the antibacterial activity of toxic compounds. On the other hand, excessive lysine can itself be a biofilm growth inhibitor itself [56]. Also, a potential decrease in the GroES cochaperone may also be a factor in biofilm growth inhibition [67,68]. Additionally, the downregulated co-chaperone GroES of *M. luteus* has 74.5% similarity with the GroS cochaperone of *Cutibacterium acnes* HL043PA2, which is one of the major protein components of the biofilm matrix of *C. acnes* HL043PA2 [69]. This finding enables us to suggest the involvement of this protein in biofilm inhibition. However, this last possibility is rather debatable because it contradicts the above-hypothesized previous version of the PNAG-mediated matrix accumulation. These processes likely exhibit considerably more complex regulation and interrelations, and there can be a dependence between surface type for cell adhesion (hydrophobic PTFE of hydrophilic glass). The downregulation of SDR oxidoreductase may hypothetically also be a factor in the decrease in biofilm cell biomass, as was shown for *H. pylori* [70].

The detection of potential epinephrine receptors in *M. luteus* cells is also of considerable interest. This bacterium had noany homologs to the QseBC/QseEF two-component systems of *E. coli*, but there is a low similarity (32.1%, e-value = 10^−37^) between histidine kinase KdpD, which has partial homology with QseC in *C. acnes* [18], and the HAMP domain containing-histidine kinase of *M. luteus* (WP_010080269.1 in the NCBI protein database). This HAMP domain containing histidine kinase does not contain EAL domains, as mentioned above. This property enables us to cautiously state that this pathway appears to be similar in at least some actinobacteria. Conversely, the hypothetical protein WP_010079694.1 is of greater interest, which has no known functions, and similarity trees built with the NCBI BLAST tool did not reveal a known protein. It is also of interest that this protein does not have conserved domains, with the exception og two EAL domains, enabling us to suggest that it belongs to the c-di-GMP phosphodiesterase according to Ref. [52]. Therefore, this finding makes allows us to hypothesize that the presence of two regulatory pathways in *M. luteus* that are sensitive to epinephrine, which warrants further investigation.

In the present study, we showed for the first time that epinephrine has a complex effect on *M. luteus* C01 isolated from human skin. There is evidence that human humoral regulation is very closely interconnected with the skin microbiota, and pathogenic microorganisms can be affected by hormones such as epinephrine. In summary, we propose a potential mechanism governing the action of epinephrine on aerobic Actinobacteria on the skin and establish a foundation for further research on microbial endocrinology.

## CRediT authorship contribution statement

**A.V. Gannesen:** Conceptualization, Methodology, Validation, Formal analysis, Investigation, Resources, Writing – original draft, Writing – review & editing, Visualization, Supervision, Project administration, Funding acquisition. **M.I. Schelkunov:** Investigation, Data curation, Formal analysis, Writing – original draft, Writing – review & editing, Visualization. **O.V. Geras'kina:** Investigation, Validation, Writing – original draft, Visualization. **N.E. Makarova:** Investigation, Validation, Writing – original draft. **M.V. Sukhacheva:** Investigation, Visualization, Validation, Writing – original draft. **N.D. Danilova:** Investigation, Writing – original draft. **M.A. Ovcharova:** Investigation, Validation. **S.V. Mart'yanov:** Methodology, Formal analysis, Investigation, Visualization, Writing – original draft, Validation, Writing – review & editing. **T.A. Pankratov:** Methodology, Investigation, Writing – original draft, Validation. **D.S. Muzychenko:** Investigation, Validation. **M.V. Zhurina:** Investigation, Validation. **A.V. Feofanov:** Validation, Writing – original draft, Writing – review & editing. **E.A. Botchkova:** Visualization, Writing – original draft, Writing – review & editing. **V.K. Plakunov:** Conceptualization, Supervision, Writing – original draft, Writing – review & editing.

## Declaration of competing interest

The authors declare that they have no known competing financial interests or personal relationships that could have appeared to influence the work reported in this paper.

## Figures and Tables

**figs1:**
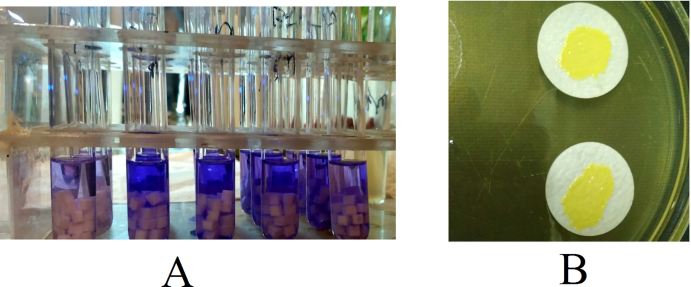


**figs2:**
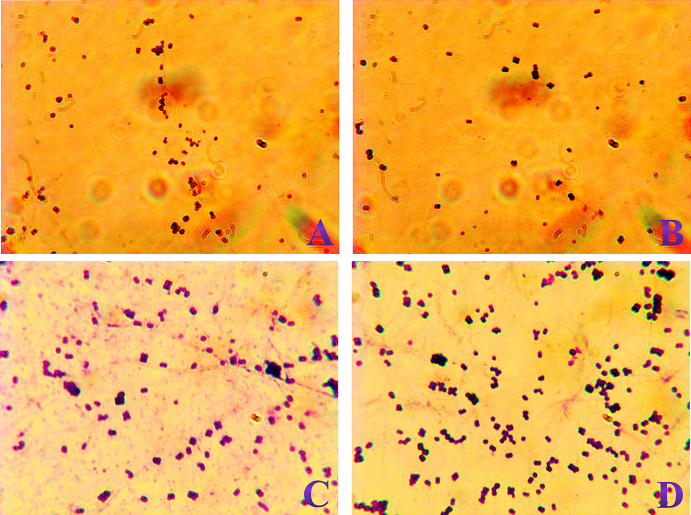


**figs3:**
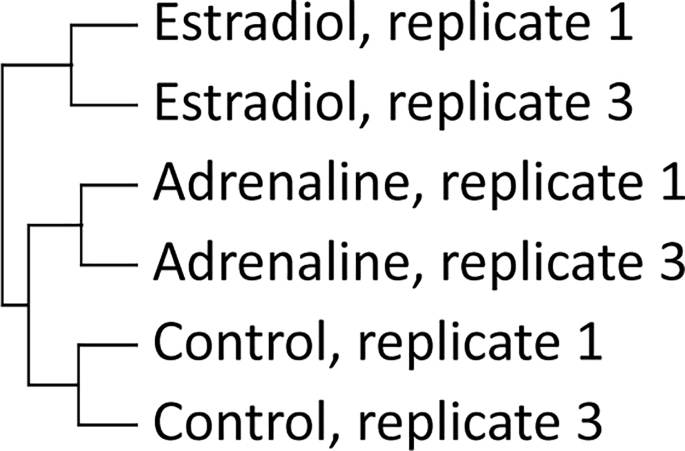


## Data Availability

The *M. luteus* C01 16S rRNA sequence was submitted to NCBI and is publicly available under the following accession code: MW167079. Data supporting the findings of this work are available within the paper and its Supplementary Information files. All other data are available from the corresponding author on request.
