# Supplementary material for: Epinephrine affects gene expression levels and has a complex effect on biofilm formation in Micrococcus luteus strain C01 isolated from human skin
Source: Biofilm. 2021 Oct 21;3:100058. doi: 10.1016/j.bioflm.2021.100058 (PMC8543384; doi:10.1016/j.bioflm.2021.100058)
Supplement: Multimedia component 1 [file mmc1.docx]

Table S1. Optical density of planktonic cultures and biofilms CV extracts after 72 h of incubation

| Planktonic cultures | | | | | | | | | |
| --- | --- | --- | --- | --- | --- | --- | --- | --- | --- |
| Control | **4.9 × 10^-12^ M** | **4.9 × 10^-11^ M** | **4.9 × 10^-10^ M** | **2.45 × 10^-9^ M** | **4.9 × 10^-9^ M** | **8.92 × 10^-9^ M** | **4.9 × 10^-8^ M** | **4.9 × 10^-7^ M** | **4.9 × 10^-6^ M** |
| 101.18 | 64.19 | 101.80 | 104.18 | 51.28 | 88.85 | 72.56 | 90.48 | 91.35 | 87.02 |
| 101.57 | 28.34 | 96.65 | 94.18 | 65.04 | 87.98 | 57.70 | 108.46 | 87.88 | 91.83 |
| 94.93 | 71.19 | 94.27 | 102.29 | 61.80 | 113.90 | 93.04 | 112.32 | 109.81 | 103.93 |
| 101.57 | 69.48 | 100.54 | 99.57 | 65.56 | 119.19 | 122.92 | 112.53 | 97.54 | 99.90 |
| 102.366 | 131.46 | 88.93 | 96.56 | 129.58 | 107.92 | 129.92 | 100.91 | 93.90 | 94.29 |
| 103.654 | 165.33 | 107.34 | 87.33 | 134.02 | 105.97 | 126.68 | 90.31 | 102.08 | 106.75 |
| 93.249 | 129.75 | 101.17 | 97.24 | 135.38 | 102.40 | 100.21 | 105.41 | 103.94 | 105.18 |
| 101.474 | 137.26 | 94.61 | 98.50 | 77.34 | 101.16 | 125.14 | 103.63 | 101.31 | 100.85 |
| 98.582 | 90.47 | 100.25 | 100.69 | 76.56 | 69.65 | 124.22 | 94.89 | 100.25 | 101.36 |
| 98.356 | 96.56 | 102.77 | 98.79 | 90.16 | 150.06 | 86.88 |  |  |  |
| 95.407 | 99.38 | 90.97 | 99.96 | 96.56 | 70.68 | 92.97 |  |  |  |
| 107.655 | 93.75 | 103.65 | 101.41 | 94.69 | 73.92 | 94.53 |  |  |  |
| 104.735 | 101.69 | 102.29 | 93.89 | 37.60 | 67.61 | 94.54 |  |  |  |
| 96.042 | 95.35 | 99.18 | 103.31 | 111.67 | 143.41 | 99.26 |  |  |  |
| 97.739 | 109.24 | 98.94 | 99.62 | 113.96 | 135.72 | 110.72 |  |  |  |
| 101.484 | 81.59 | 89.90 | 99.42 | 91.30 | 89.06 | 109.37 |  |  |  |
| 102.073 | 81.70 | 100.10 | 105.59 | 84.62 | 84.84 | 101.44 |  |  |  |
| 103.29 | 83.82 | 100.10 | 94.81 | 113.30 | 110.31 | 99.76 |  |  |  |
| 97.64 | 85.42 |  |  | 119.94 | 137.50 | 103.92 |  |  |  |
| 96.99 | 97.19 |  |  | 81.52 | 96.83 | 82.76 |  |  |  |
| 110.90 | 81.98 |  |  | 104.09 | 96.48 | 77.68 |  |  |  |
| 107.207 | 122.32 |  |  | 113.12 |  | 111.05 |  |  |  |
| 83.423 | 106.84 |  |  | 105.55 |  | 81.46 |  |  |  |
| 98.468 | 107.10 |  |  | 89.37 |  | 106.84 |  |  |  |
|  |  |  |  |  |  |  |  |  |  |
|  |  |  |  |  |  |  |  |  |  |
| Biofilms | | | | | | | | | |
| Control | **4.9 × 10^-12^ M** | **4.9 × 10^-11^ M** | **4.9 × 10^-10^ M** | **2.45 × 10^-9^ M** | **4.9 × 10^-9^ M** | **8.92 × 10^-9^ M** | **4.9 × 10^-8^ M** | **4.9 × 10^-7^ M** | **4.9 × 10^-6^ M** |
| 91.87 | 103.47 | 86.14 | 94.34 | 82.67 | 113.40 | 88.00 | 99.04 | 114.83 | 106.22 |
| 132.06 | 81.07 | 100.18 | 89.99 | 101.33 | 114.83 | 65.07 | 159.33 | 97.61 | 67.46 |
| 76.08 | 72.00 | 98.50 | 88.19 | 58.13 | 166.00 | 59.73 | 126.00 | 130.00 | 118.00 |
| 80.00 | 133.87 | 100.37 | 97.63 | 120.00 | 226.00 | 67.73 | 104.00 | 90.00 | 134.00 |
| 66.00 | 41.06 | 92.60 | 95.46 | 44.70 | 232.94 | 72.52 | 188.40 | 70.14 | 100.34 |
| 154.00 | 61.92 | 96.58 | 108.26 | 69.54 | 212.97 | 89.07 | 194.54 | 173.55 | 89.08 |
| 129.52 | 100.15 | 93.47 | 95.77 | 83.00 | 238.48 | 71.36 | 89.09 | 67.27 | 106.67 |
| 94.71 | 79.94 | 101.86 | 99.13 | 43.80 | 119.47 | 37.37 | 100.30 | 68.79 | 113.33 |
| 75.77 | 44.87 | 87.07 | 88.93 | 239.74 | 98.07 | 103.85 | 97.82 | 80.12 | 104.89 |
| 104.85 | 305.77 | 99.81 | 97.82 | 141.03 | 123.20 | 193.59 |  |  |  |
| 106.67 | 260.00 | 96.08 | 90.11 | 235.00 | 59.73 | 187.50 |  |  |  |
| 88.48 | 362.50 | 100.06 | 90.30 |  | 73.18 |  |  |  |  |
| 43.1 |  | 99.19 | 96.89 |  | 63.25 |  |  |  |  |
| 130.7 |  | 100.62 | 89.00 |  | 97.09 |  |  |  |  |
| 107.76 |  | 92.10 | 96.08 |  | 194.87 |  |  |  |  |
| 132.79 |  | 100.56 | 103.79 |  | 278.2 |  |  |  |  |
| 76.4774 |  | 99.69 | 93.22 |  | 212.5 |  |  |  |  |
| 109.154 |  | 103.17 | 92.35 |  | 337.5 |  |  |  |  |
| 91.87 |  |  |  |  | 125.84 |  |  |  |  |
| 132.06 |  |  |  |  | 142.5261 |  |  |  |  |
|  |  |  |  |  | 193.97 |  |  |  |  |
|  |  |  |  |  | 66.1 |  |  |  |  |
|  |  |  |  |  | 95.2 |  |  |  |  |
|  |  |  |  |  | 186.3 |  |  |  |  |
|  |  |  |  |  | 113.40 |  |  |  |  |
|  |  |  |  |  | 114.83 |  |  |  |  |
