# Supplementary material for: Epinephrine affects gene expression levels and has a complex effect on biofilm formation in Micrococcus luteus strain C01 isolated from human skin
Source: Biofilm. 2021 Oct 21;3:100058. doi: 10.1016/j.bioflm.2021.100058 (PMC8543384; doi:10.1016/j.bioflm.2021.100058)
Supplement: Multimedia component 2 [file mmc2.docx]

Table S2. CFU amount in *M. luteus* C01 biofilms

|  | Control 24h | | Epinephrine 24 h | | Control 72 h | | Epinephrine 72 h | |
| --- | --- | --- | --- | --- | --- | --- | --- | --- |
|  | **Absolute** | **%** | **Absolute** | **%** | **Absolute** | **%** | **Absolute** | **%** |
| Repeat 1 | 3.8 × 10^8^ | 131.8 | 2.8 × 10^8^ | 97.2 | 3.4 × 10^9^ | 55.1 | 4.5 × 10^9^ | 72. 9 |
| Repeat 2 | 2.8 × 10^8^ | 97.6 | 1.9 × 10^8^ | 64.2 | 3.8 × 10^9^ | 61.5 | 6.2 × 10^9^ | 100.4 |
| Repeat 3 | 3.1 × 10^8^ | 106.6 | 1.9 × 10^8^ | 67.8 | 7.7 × 10^9^ | 124.7 | 4.6 × 10^9^ | 74.5 |
| Repeat 4 | 1.8 × 10^8^ | 64.0 | 1.3 × 10^8^ | 43.9 | 9.8 × 10^9^ | 158.7 | 6.4 × 10^9^ | 103.6 |
|  |  |  |  |  |  |  |  |  |
| Average | 2.9 × 10^8^ | 100 | 2 × 10^8^ | 68.3 | 6.2 × 10^9^ | 100 | 5.4 × 10^9^ | 87.9 |
| St.dev. | 8.0 × 10^7^ | 28.0 | 6.3 × 10^7^ | 21.0 | 3.1 × 10^9^ | 50.2 | 1.0 × 10^9^ | 16.4 |
| SEM | 4.0 × 10^7^ | 14.0 | 3.2х10^7^ | 11.0 | 1.0 × 10^9^ | 16.7 | 3.4 × 10^8^ | 5.5 |
