# Supplementary material for: Epinephrine affects gene expression levels and has a complex effect on biofilm formation in Micrococcus luteus strain C01 isolated from human skin
Source: Biofilm. 2021 Oct 21;3:100058. doi: 10.1016/j.bioflm.2021.100058 (PMC8543384; doi:10.1016/j.bioflm.2021.100058)
Supplement: Multimedia component 3 [file mmc3.docx]

Table S3. Primers used in RT-PCR experiments.

| Gene № | Gene name and product | Sequence |
| --- | --- | --- |
| 1 | MLUT_RS12070  SDR family oxidoreductase | F 5’-GTCGTGTTCCGTCTGAAGTC-3’ |
|  |  | R 5’-GTCGTTCAGCGCGCCCGC-3’ |
| 2 | MLUT_RS12075  hypothetical protein | F 5’-AGGGCACCTTCGTGATGT-3’ |
|  |  | R 5’-ATGTCGCAGCGCATCTCCTC-3’ |
| 3 | MLUT_RS19770  co-chaperone GroES | F 5’-GACACCGCCAAGGAGAAG-3’ |
|  |  | R 5’-CCTCGGTGCCGCCGTACTT-3’ |
| 4 | MLUT_RS22450  LysE family transporter | F 5’-CACCGGATTCCTGACCTAC-3’ |
|  |  | R 5’-AGCAGCGCCACATCGGCC-3’ |
| 5 | MLUT_RS23355  VIT1/CCC1 transporter family protein | F 5’-GAGTACGTGTCCGTCTCCTC-3’ |
|  |  | R 5’-CCGTGGCCCGGCTCAGGCCG-3’ |
| 6 | MLUT_RS16880  sulfurtransferase | F 5’-AGGACGCTCTCCTCTACTCC-3’ |
|  |  | R 5’-GGCGGCCCACCAGTTGGAC-3’ |
| 7 | MLUT_RS16875  SufE family protein | F 5’-TCGAGCTGCTGCTGGAGT-3’ |
|  |  | R 5’-CCCTCGAGCTCGACGGCC-3’ |
| 8 | MLUT_RS17260  Fe-S cluster assembly protein SufB | F 5’-CTCGTACGTGCACTACATCG-3’ |
|  |  | R 5’-TGTACACGTTCGTGGACCAG-3’ |
| 9 | MLUT_RS17265  Fe-S cluster assembly protein SufD | F 5’-ACGGACACGTACGAGAAGAA-3’ |
|  |  | R 5’-GATGCCGCGGGCCATCAGGT-3’ |
| 10 | MLUT_RS17275  metal-sulfur cluster assembly factor | F 5’-GCTCTACGGGCTGCACTAC-3’ |
|  |  | R 5’-CTCGGGGCCCCACGGGGG-3’ |
| 11 | MLUT_08120 ref  “A” region of the F0 subunit of a conserved ATP synthase | F 5’-GCGAGAAGAACTTCAAGCAG-3’ |
|  |  | R 5’-TTGATGCCGACGCCGATCC-3’ |
|  |  |  |
