# Supplementary material for: Epinephrine affects gene expression levels and has a complex effect on biofilm formation in Micrococcus luteus strain C01 isolated from human skin
Source: Biofilm. 2021 Oct 21;3:100058. doi: 10.1016/j.bioflm.2021.100058 (PMC8543384; doi:10.1016/j.bioflm.2021.100058)
Supplement: Multimedia component 4 [file mmc4.zip › bioflm_100058_Suppl Table 4 R2_mmc4.DOC]

Table S4. Analysis of the contamination of the total RNA samples

|  | **Family** | **The number of reads with the best match to a sequence from this family** |
| --- | --- | --- |
|  | *M. luteus* control replicate 1 | |
|  | *Micrococcaceae* | 986 |
|  | No similar sequences | 6 |
|  | *Pseudomonadaceae* | 4 |
|  | *Propionibacteriaceae* | 1 |
|  | No similar sequences | 1 |
|  | *Microbacteriaceae* | 1 |
|  | *Hominidae* | 1 |
|  |  |  |

|  | *M. luteus* control replicate 2 |
| --- | --- |
| *Micrococcaceae* | 992 |
| No similar sequences | 2 |
| *Dermatophilaceae* | 2 |
| *Moraxellaceae* | 1 |
| *Microbacteriaceae* | 1 |
| Unknown family | 1 |
| *Pseudomonadaceae* | 1 |
|  |  |

|  | *M. luteus* epinephrine 4.9 × 10^-9^ M replicate 1 | |
| --- | --- | --- |
| *Micrococcaceae* |  | 984 |
| *No similar sequences* |  | 4 |
|  |  |  |
| *Pseudomonadaceae* |  | 4 |
| *Nocardiaceae* |  | 4 |
| *Microbacteriaceae* |  | 2 |
| *Cellulomonadaceae* |  | 1 |
| *Actinomycetaceae* |  | 1 |
|  |  |  |

|  | *M. luteus* epinephrine 4.9 × 10^-9^ M replicate 2 | |
| --- | --- | --- |
| *Micrococcaceae* |  | 977 |
| No similar sequences |  | 7 |
| *Mycobacteriaceae* |  | 3 |
| Unknown family |  | 3 |
| *Dermatophilaceae* |  | 3 |
| *Microbacteriaceae* |  | 2 |
| *Pseudomonadaceae* |  | 2 |
| *Streptomycetaceae* |  | 2 |
| *Brevibacteriaceae* |  | 1 |
|  |  |  |
